# Impact of Hypertension and Physical Exercise on Hemolysis Risk in the Left Coronary Artery: A Computational Fluid Dynamics Analysis

**DOI:** 10.3390/jcm13206163

**Published:** 2024-10-16

**Authors:** Krystian Jędrzejczak, Wojciech Orciuch, Krzysztof Wojtas, Piotr Piasecki, Jerzy Narloch, Marek Wierzbicki, Michał Kozłowski, Malenka M. Bissell, Łukasz Makowski

**Affiliations:** 1 Faculty of Chemical and Process Engineering, Warsaw University of Technology, Waryńskiego 1, 00-645 Warsaw, Poland; 2 Leeds Institute of Cardiovascular and Metabolic Medicine, University of Leeds, Leeds LS2 9NL, UK; 3 Interventional Radiology Department, Military Institute of Medicine-National Research Institute, Szaserów 128, 04-141 Warsaw, Poland; 4 Department of Cardiology and Structural Heart Diseases, Medical University of Silesia, Ziołowa 47, 40-635 Katowice, Poland

**Keywords:** hypertension, coronary artery disease, atherosclerosis, blood, computational fluid dynamics, fluid–structure interactions

## Abstract

**Background and Objectives**: Hypertension increases the risk of developing atherosclerosis and arterial stiffness, with secondarily enhanced wall stress pressure that damages the artery wall. The coexistence of atherosclerosis and hypertension leads to artery stenosis and microvascular angiopathies, during which the intravascular mechanical hemolysis of red blood cells (RBCs) occurs, leading to increased platelet activation, dysfunction of the endothelium and smooth muscle cells due to a decrease in nitric oxide, and the direct harmful effects of hemoglobin and iron released from the red blood cells. This study analyzed the impact of hypertension and physical exercise on the risk of hemolysis in the left coronary artery. **Methods**: To analyze many different cases and consider the decrease in flow through narrowed arteries, a flow model was adopted that considered hydraulic resistance in the distal section, which depended on the conditions of hypertension and exercise. The commercial ANSYS Fluent 2023R2 software supplemented with user-defined functions was used for the simulation. CFD simulations were performed and compared with the FSI simulation results. **Results**: The differences obtained between the FSI and CFD simulations were negligible, which allowed the continuation of analyses based only on CFD simulations. The drops in pressure and the risk of hemolysis increased dramatically with increased flow associated with increased exercise. A relationship was observed between the increase in blood pressure and hypertension, but in this case, the increase in blood pressure dropped, and the risk of hemolysis was not so substantial. However, by far, the case of increased physical activity with hypertension had the highest risk of hemolysis, which is associated with an increased risk of clot formation that can block distal arteries and lead to myocardial hypoxia. **Conclusions**: The influence of hypertension and increased physical exercise on the increased risk of hemolysis has been demonstrated.

## 1. Introduction

Coronary artery disease (CAD) has become increasingly prevalent in the 21st century, driven by lifestyle factors and aging populations [1,2,3,4,5]. Most often, it is a consequence of coronary atherosclerosis. The development of atherosclerosis is multifactorial and is primarily related to advanced age, smoking, chronic hypertension, diabetes, and genetic predisposition. Environmental factors like an unhealthy diet, lack of physical activity, and smoking significantly contribute to the development and progression of atherosclerosis [6,7]. Constantly increasing incidence rates of cardiovascular diseases create a need for the development of modern non-invasive diagnostic techniques of the vascular system. Atheromatous plaques are raised, which are focal lesions within the intima of the artery wall. They are composed of a soft necrotic core (lipids, foam cells) covered by a fibrous/calcified cap. Atherosclerotic plaques are most prominent at artery bifurcation points and the ostia of major branches. Advanced plaques decrease the lumen of the coronary arteries, leading to critical stenosis, reduced blood flow, and potential myocardial hypoxia. Moreover, arterial stenosis disturbs laminar blood flow and leads to the turbulence of blood morphological elements, which increases the risk of mechanical hemolysis. In the case of narrowed arteries, severe hemolysis can exacerbate myocardial hypoxia, particularly in the presence of vasostenosis; additionally, released coagulation factors may increase the risk of clot formation. Moreover, increased local velocity in the stenosis area increases the turbulence in the area after stenosis; the local jet which originates from stenosis can cause local recirculation zones or dead zones. In these zones, red blood cells which were previously destroyed because of higher shear stress in the stenosis zone can release Fe^2+^ cations and toxic hemoglobin, which can stay in the recirculation zone or dead zone long enough to cause the formulation of a clot [8,9]. Finally, intravascular hemolysis, disruption of the atherosclerotic plaque, and other factors lead to acute coronary syndromes.

Hypertension is a strong risk factor for cardiovascular diseases, and it is closely associated with coronary artery disease (CAD). The simultaneous occurrence of arterial hypertension and atherosclerosis is a continuous relationship between an increase in blood pressure (BP) and the risk of stroke, coronary artery disease (CAD), heart failure (HF), and the development and progression of chronic kidney disease (CKD) [10,11]. A recent meta-analysis suggests that for every 10 mmHg reduction in systolic blood pressure, CAD can be reduced by 17% [12]. Regular moderate submaximal physical activity is recommended at least once a week for the primary prevention of cardiovascular diseases. However, in patients diagnosed with uncomplicated coronary artery disease or after a myocardial infarction, starting a hard, exhausting maximum physical exercise programme in previously inactive people may increase the risk of acute coronary syndrome.

This study is focused on the impact of hypertension and intense physical exercise in previously inactive people on the risk of complications in coronary artery disease. First, the influence of blood vessel deformation on pressure drops was checked by comparing the results of Fluid–Structure Interaction (FSI) simulations with CFD simulations. CFD simulations were then performed for various locations and degrees of atherosclerotic stenosis. Simulations were conducted under varying conditions, including different levels of physical activity, hypertension, and normal blood pressure. The results obtained were collected as bar charts for later analysis. This study aims to provide insights into the cardiovascular risks associated with hypertension and exercise in CAD patients, potentially guiding more effective prevention strategies.

## 2. Materials and Methods

### 2.1. Geometries

**Figure 1:**
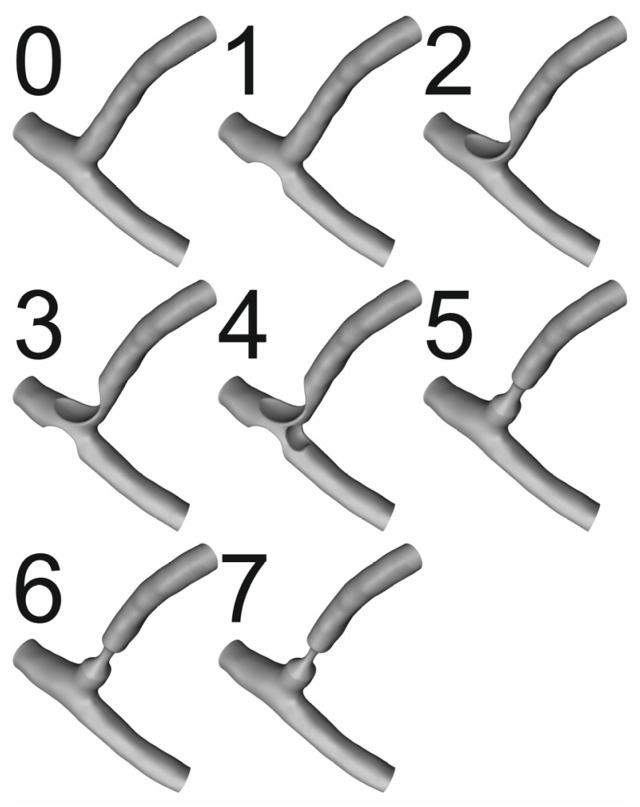
Artery geometry variants.

Figure 1 shows a series of geometries of the left coronary artery and the left anterior descending and left circumflex artery. Variant 0 shows healthy arteries with no signs of atherosclerosis. However, variants 1 to 4 represent different variants of atherosclerosis near the bifurcation. Variants 5 to 7 correspond to the narrowing of the left circumflex artery to 50%, 40%, and 30% of the diameter of the healthy artery, respectively. Atherosclerosis plaque is predominantly located in the lateral walls of the bifurcations, side branches, and arteries ostia. The most common location is the left coronary artery. Specifically, the highest percentage of plaque deposits are found in the left anterior descending artery (LAD), followed by the right coronary artery (RCA), circumflex branch (CX), and left main stem (LM) [13,14,15,16,17,18].

### 2.2. Rheology and Hemolysis

The blood viscosity model is based on population balance. Details related to population balance-based rheology (PBBR) were presented in previous publications [19,20,21]. The model predicts the agglomeration of red blood cells in areas with low shear rates and deagglomerations in regions with high shear rates. Consequently, PBBR predicts the physiological distribution of the size of red blood cell agglomerates, which can be observed across the cross-section of the vessel. As a result, the viscosity is greatest on the axis of the vessel.

Furthermore, the thixotropic effects of blood [22,23,24,25] were also observed. The hemolysis model is based on the power law model and its later alterations [26,27,28]. The PBBR model refers to the hemolysis of only fully deagglomerated single red blood cells, whose concentration is calculated using the Direct Quadrate Method of Moments (DQMOM) [19]. The DQMOM is a convenient method for solving the system of population balance equations using discrete representations of weights and abscissas without the need to reconstruct discrete population balance distribution compared to the Quadrate Method of Moments (QMOM).

(1) $$\Delta H_{b}/H_{b}=\frac{H\cdot m_{s}L_{0}^{3}\;}{m_{s}L_{0}^{3}+\sum_{i=1}^{3}w_{i}L_{i}^{3}}$$

where $m_{s}$ is the concentration of single red blood cells in the DQMOM, $L_{i}$ are the sizes of the agglomerates used in DQMOM, and $w_{i}$ are the weights used in the DQMOM.

### 2.3. Numerical Settings

**Figure 2:**
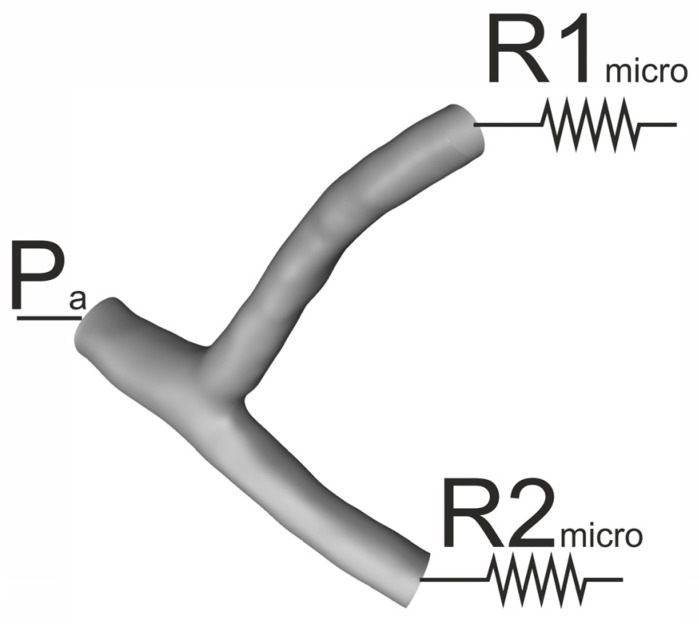
Boundary condition schematic.

The calculations were performed using ANSYS Fluent 2023R2 software supplemented with user-defined functions. The population balance was solved using the DQMOM method, which was implemented by user-defined functions. The generalized k-ω (GEKO) turbulence model with the option to model the transient range enabled was chosen because turbulent flow occurred locally in the area of stenosis. Average blood flow values in a steady state were used to simplify the analysis. In the case of FSI simulations, the mesh was tetrahedral, while for further CFD simulations, the mesh was polyhedral. This action was due to the incompatibility of the FSI module with the polyhedral mesh. A mesh independence test was also performed to select a mesh with appropriate density. To adequately represent the pressure drop in the area of the coronary vessels, it was assumed that the static pressure at the inlet would be constant and that a change in the pressure distribution in the coronary vessels would not significantly affect the pressure in the aorta. However, to properly account for the pressure drop in further sections of the coronary vessels, a constant hydraulic resistance was assumed, which was selected to ensure the physiological blood flow for variant 0, corresponding to healthy arteries. The relationships describing the outlet pressure based on hydraulic resistance are presented in Equations (2) and (3). In the absence of hypertension, the pressure at the inlet to the left coronary artery was assumed to be 93 mmHg and 130 mmHg in the case of hypertension stage 2 [29]. The boundary conditions for pressure are symbolically summarized in Figure 2.

(2) $$P_{LCX}=R1_{micro}\cdot Q_{LCX}$$

(3) $$P_{LAD}=R2_{micro}\cdot Q_{LAD}$$

The values of hydraulic resistance are summarized in Table 1.

In the case of walls, a no-slip condition was assumed.

Moreover, the size distribution of red blood cell agglomerates at the inlet was assumed to be an approximate solution of the population balance for viscosimetric conditions as a function of the local shear rate for cells directly at the inlet to the system. In the case of CFD simulations, the deformation of the vessel under the influence of pressure was not considered; however, in the case of FSI simulations, the Intrinsic FSI condition was used for walls directly in contact with blood. However, no displacement was assumed for the walls at the inlet and outlets, and the Stress-Free condition was considered for the remaining external walls. For FSI simulation, a dynamic mesh with the smoothing option and the Linearly Elastic Solid suboption was enabled. The vessel wall density was assumed to be 1120 kg/m^3^, Young’s modulus was 1.5 MPa, and the Poisson ratio was 0.49.

## 3. Results

### 3.1. FSI Simulation

FSI simulations were performed to check whether the mesh deformation would be significant in terms of changes in pressure drop.

**Figure 3:**
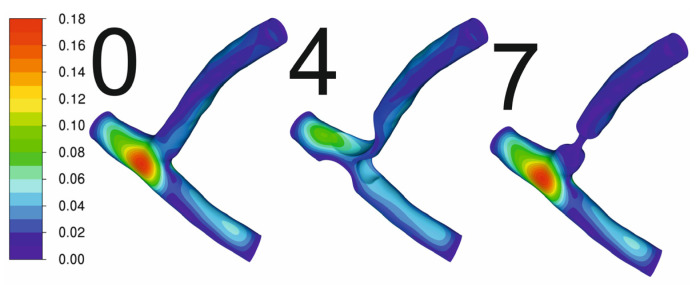
Displacement [mm] of the geometry walls from FSI simulation.

Figure 3 shows the deformation of the artery geometry for variants 0, 4, and 7. These deformations are relatively small in terms of the size of the geometry. The deformations in the area of stenosis are much smaller than in the case of a healthy artery, which is a consequence of the much thicker vessel wall in the area of stenosis. FSI simulations can provide information about deformations of the artery wall and affect the results; however, FSI simulations require higher computing power and are more time-consuming. The steady-state simulations of coronary artery deformations caused by pressure differences are insignificant compared to the potential movement caused by heart work itself. FSI simulations can provide more information where the aorta is taken into consideration, and the deformation of the aorta wall can be seen as a result of rapid pressure changes. Nevertheless, CFD simulations can provide accurate results for coronary arteries because deformations are negligible as, when comparing the results of the FSI simulations with the CFD simulations, the difference in pressure drop did not exceed 0.2%. Therefore, the rest of this research focused on using only CFD simulations.

### 3.2. CFD Results

CFD simulations were performed for all geometric variants and combinations of boundary conditions. 

**Figure 4:**
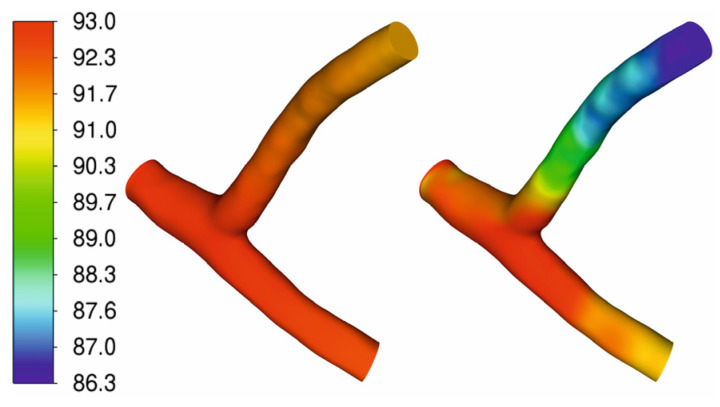
Contour plots of static pressure [mmHg] for rest (**left**) and exercise (**right**).

Figure 4 shows the static pressure distribution [mmHg] during rest and sudden physical exercise. It can be noted that in the case of exercise, the pressure drop is much more significant. However, despite intense workouts, the pressure drop is only a few mmHg.

**Figure 5:**
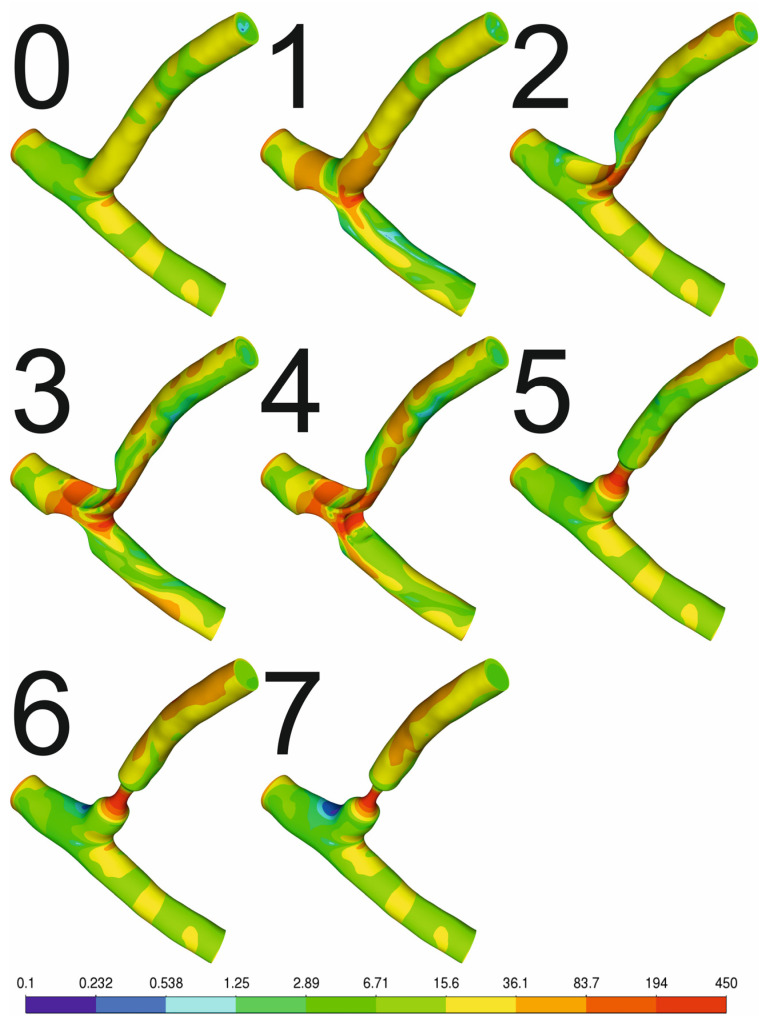
Contour plots of shear stress [Pa] for exercise without hypertension.

Figure 5 shows the shear stress distribution for all geometric variants for exercise without hypertension. In the case of a healthy artery, there are no areas where shear stresses exceed 150 Pa, which could result in hemolysis. However, the greater the degree of atherosclerosis, the greater the shear stresses, which is especially visible for variants 3–7. The risk of hemolysis is significant in the case of such a large amount of atherosclerotic plaque deposits. Patients with lesions 2–7 may have significantly limited physical capacity (i.e., difficulty climbing more than two floors without stopping) and are unable to perform moderate-to-heavy physical activity. Patients with such stenoses have a high risk of acute coronary syndrome.

**Figure 6:**
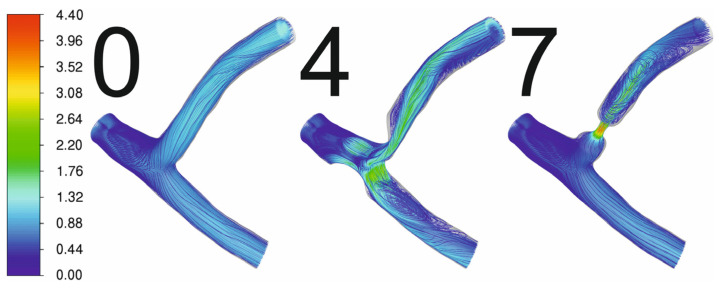
Pathlines for variants 0, 4, and 7 coloured by velocity magnitude [m/s].

Figure 6 shows the pathlines for variants 0, 4, and 7. In the case of arteries with atherosclerotic stenosis, rapid accelerations of fluid elements occur, and recirculation zones with high vorticity are formed behind the narrowing. These zones are at an increased risk of a clot forming, which can block the arteries. During initial angiography before the angioplasty of arterial stenoses, turbulence and flow stagnation are observed behind the stenoses, and are especially visible at critical stenoses of approximately 90%. Furthermore, due to this volume overload phenomenon, the dilation of the arteries behind the stenoses occurs, which is called “post-stenotic arterial dilatation”.

Normalized pressure is defined as a ratio of static pressure at the outlet of the vessel to static pressure at the inlet to the left main coronary artery, which is assumed to be equal to aortic pressure.

**Figure 7:**
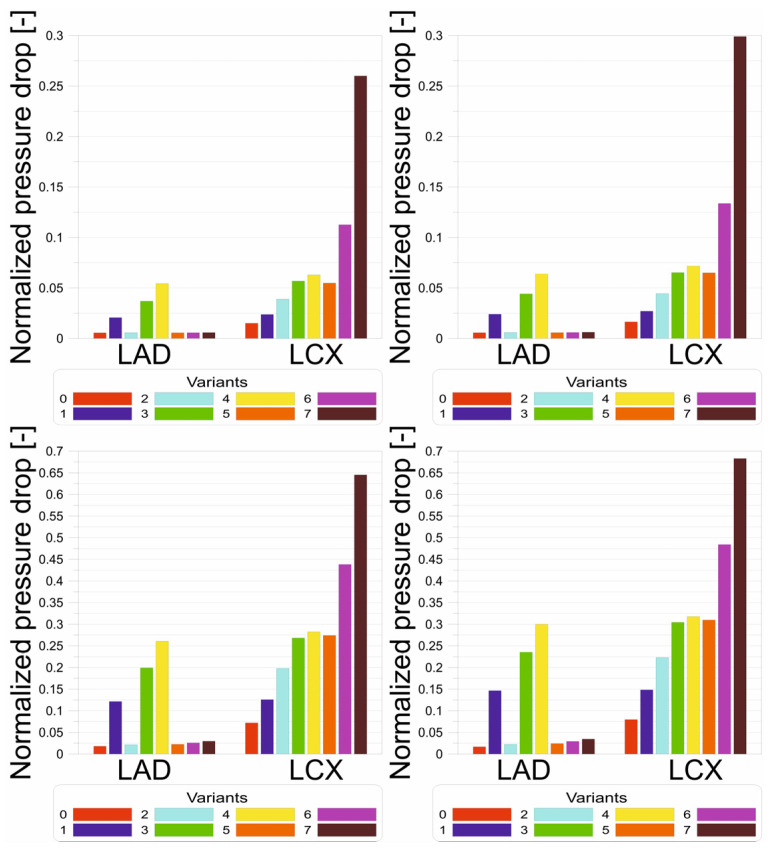
Normalized pressure [-] bar charts for (**top left**) rest without hypertension, (**top right**) rest with hypertension, (**bottom left**) exercise without hypertension, and (**bottom right**) exercise with hypertension.

Figure 7 shows the results of the normalized pressure drop at the outlets from LAD and LCX. It can be seen that hypertension has a negligible effect on increasing the pressure drop for all analyzed cases. However, when activity increases, the pressure drop increases dramatically. Without exercise, the pressure drop is only higher than 10% for free lumen below 40% LCX diameter. However, in the case of exercise, the pressure drop can reach up to 70% percent for variant 7. It is worth noting that the pressure drop is small regardless of the conditions for healthy arteries without any cholesterol deposits. The results indicate that FFR measurements after adenosine administration are more sensitive than CRR measurements without adenosine. However, this also proves that the test with adenosine administration is associated with a more significant burden on the body. During exercise and an increase in systemic blood pressure, the heart’s afterload is increased, extending the systolic phase during which the flow through the coronary vessels is limited. The decrease in coronary blood flow (initially reduced by coronary artery stenosis) may worsen during exercise. This phenomenon is used to assess the clinical significance of stenoses in exercise tests. We then obtain an image of ischemia in the ECG recording; the patient may present with typical coronary pain or stop exercising due to rapid fatigue.

**Figure 8:**
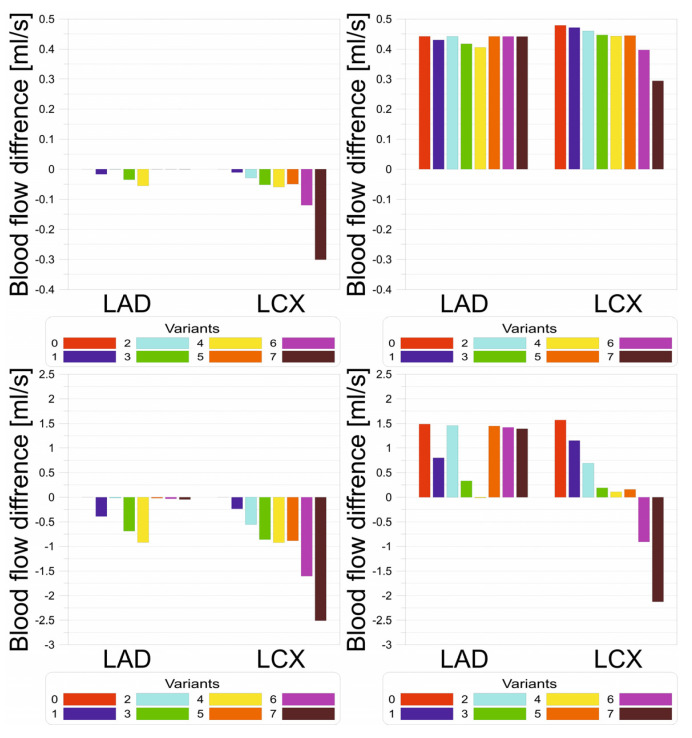
Volume flow rate [mL/s] bar charts for (**top left**) rest without hypertension, (**top right**) rest with hypertension, (**bottom left**) exercise without hypertension, and (**bottom right**) exercise with hypertension.

Figure 8 shows a blood volume flow difference compared to healthy patients without atherosclerosis and hypertension during rest and exercise for all analyzed atherosclerotic constriction geometry variants. It can be seen that hypertension causes an increase in blood flow through the vessel for both the rest and exercise conditions. This increase is visible even in cases of severe cholesterol narrowing during rest; however, during exercise, severe atherosclerosis causes a decrease in blood flow greater than the increase generated by hypertension. It is worth noting that the same narrowing of the artery during rest may be completely harmless but very dangerous during rapid and intense exercise. During exercise, however, the demand for oxygen in the heart muscle is higher and there may be an insufficient increase in blood flow, resulting in an inadequate rise in oxygen transport to the muscle with regard to needs. This situation may lead to the risk of myocardial hypoxia.

Arterial hypertension is a stable, chronic clinical condition to which patients’ systems, including the cardiovascular system, adapt, maintaining the flow in the organs at a relatively constant level.

During physical exercise, the oxygen demand peripherally increases, including in the muscles, which is why the heart begins to perform excessive contractile work, which subsequently reduces the flow in the coronary arteries (the greatest flow in the coronary arteries is observed during diastole of the heart—during exercise, the diastolic phase is shortened). This results in a reduction in coronary blood flow, leading to hypoxia in patients with narrowing of the coronary arteries. Such a patient may end exercise prematurely due to fatigue and/or chest pain. In addition, the patient may develop an early and long-term elevated heart rate and deep rapid breathing. Long-term heavy physical exercise in patients with significant or critical stenoses may cause acute coronary syndrome.

**Figure 9:**
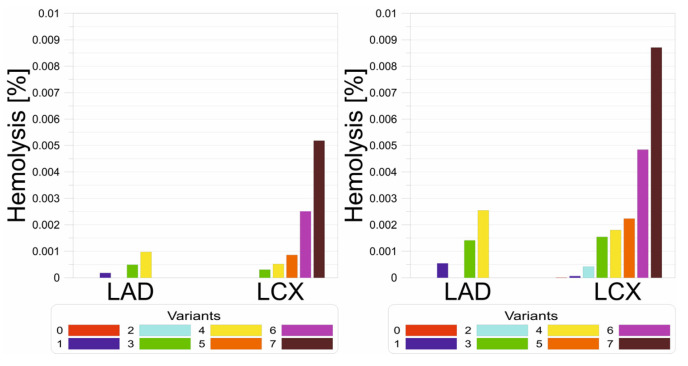
Hemolysis [%] bar charts for (**left**) exercise without hypertension and (**right**) exercise with hypertension.

Figure 9 compares the intensity of hemolysis in the case of exercise with and without hypertension. It can be seen that the greater the degree of atherosclerotic narrowing, the more intense the hemolysis. It is worth noting that in the case of hypertension, hemolysis is dramatically multiplied, and is associated with greater blood flow, which is possible due to higher aortic pressure. The obtained results indicate that simultaneous atherosclerosis and hypertension multiply the risk of hemolysis, which is associated with an increased risk of clot formation, which may block the distal section of the artery, resulting in myocardial infarction.

## 4. Discussion

Hypertension is characterized by consistently high blood pressure in the body’s arteries and is the most significant risk factor for cardiovascular disease and overall mortality worldwide. The majority of hypertension cases are essential or primary, with an unknown cause, while about 10% are secondary, with an identifiable cause. The development of hypertension involves complex interactions among environmental and behavioural factors, genetic and hormonal networks, and various organ systems such as the renal, cardiovascular, and central nervous systems. Additionally, vascular and immune mechanisms play a role. The dysregulation of these processes leads to hypertension, which, if left uncontrolled, can result in organ damage and negative cardiovascular outcomes. The key issue is appropriate prevention and early diagnosis to stop the development of diseases. A proper diet and physical activity limit the development of diseases. It is important to adjust increases in physical activity in relation to the current physical condition, as a sudden increase in activity may cause negative effects in people with advanced hypertension and atherosclerosis.

A detailed understanding of the relationship between atherosclerosis, hypertension, and physical activity will allow for improved diagnosis of the above diseases and the development of guidelines for better treatment planning. The knowledge of stenosis-related hemolysis in general practitioners is lacking, since there is no diagnostic test to evaluate it, i.e., in regular blood tests. Despite proper guideline-backed treatment, there are cases of disease exacerbation, often related to exercise. The results of CFD simulations indicate that atherosclerosis combined with arterial hypertension results in a greater pressure difference between the aorta and the distal section of the artery, maintaining a greater blood flow in contrast to atherosclerosis, where a smaller bloodstream may flow through the narrowed vessels due to the significant pressure drop at the atherosclerotic stenosis. The increase in flow in the vessel caused by hypertension, on the one hand, indicates a lower risk of hypoxia in further parts of the circulatory system. On the other hand, the higher flow causes higher shear stresses, which significantly increase the risk of platelet activation, alteration of the coagulation cascade, thrombosis and emboli. Shear stress elevation can lead to the destruction of red blood cells, which leads to the dysfunction of the endothelium and smooth muscle cells due to a decrease in nitric oxide and the direct harmful effects of hemoglobin and iron released from the red blood cells. In combination with intense physical exercise, where the heart pumps more blood, there is a significant increase in shear stress and the risk of hemolysis, which may strongly affect the risk of clot formation, which will block the distal section of the artery, causing a risk of myocardial hypoxia as a result of intense physical activity or mechanical trauma. The simulation results suggest that for people with severe atherosclerosis and hypertension, physical activity should be increased gradually because a sudden increase in physical activity may pose a serious risk of embolism. Some patients may benefit from more aggressive antiplatelet treatment or additional coronary circulation evaluation, i.e., FFR.

Currently, a commonly used test in the diagnosis of atherosclerosis is the measurement of FFR after prior administration of adenosine [30,31,32,33]. This examination is often accompanied by arterial angiography. If a patient is diagnosed with atherosclerosis, the most common treatment method is Percutaneous Coronary Intervention (PCI) [34,35,36,37], where appropriately adjusted stents are used. These stents may be coated with a drug (Drug-Eluting Stents) that reduces the risk of restenosis [38]. Due to the influence of hypertension and physical activity on the risk of hemolysis, the phenomenon of restenosis is additionally dangerous for the patient because the complications are more serious. The use of Drug-Eluting Stents may reduce this risk. In the period after the procedure, it is important to follow a healthy diet and change one’s lifestyle to gradually increase physical activity, as it reduces the risk of atherosclerosis. Increasing physical activity later on after restenosis occurs is difficult because the body’s efficiency decreases and the risk of embolism increases; hence, the patient needs to take build their fitness early on.

This study’s main limitation is that its considerations are limited to the section of the left coronary artery with a bifurcation into the left circumflex artery and the descending artery. It was assumed that the flow in the left artery did not influence the blood flow in the aorta. Moreover, the average flow was assumed to be in a steady state to simplify the analysis and observe general regularities. A possible next stage of research would be to take into account the geometry of the aorta and right coronary artery in an unsteady state, account for arterial compliance where the blood flow from the heart would be set, and as a consequence of taking into account the stenosis in the coronary arteries, the blood flow in the coronary arteries would be reduced, and on the aorta’s outlet it will rise. In order to enhance the model and analyze its sensitivity to geometry, it may be beneficial to take patient-specific configurations into account.

## 5. Conclusions

Advanced coronary atherosclerosis and hypertension co-occurrence significantly increase the risk of acute coronary syndrome and worsen its prognosis. Performing maximum physical exertion in patients with untreated coronary artery disease may cause an acute coronary event. Our results may indicate that FFR measurements with adenosine administration are more sensitive to pressure changes but are associated with a more significant reduction in coronary blood flow and increased myocardial hypoxia. FFR evaluation might improve patient selection for angioplasty in those planning on more physical activity in the future.

## Figures and Tables

**Table 1:** Hydraulic resistance and healthy blood flow for LAD and LCX arteries.

	$\begin{array}{c} R1_{micro}\\ \left[kg\cdot m^{-4}\cdot s^{-1}\right] \end{array}$	$\begin{array}{c} R2_{micro}\\ \left[kg\cdot m^{-4}\cdot s^{-1}\right] \end{array}$
Rest	$1.01\cdot 10^{10}$	$1.11\cdot 10^{10}$
Exercise	$2.83\cdot 10^{9}$	$3.27\cdot 10^{9}$
	$Q_{1}\;[m^{3}\cdot s^{-1}]$	$Q_{2}\;[m^{3}\cdot s^{-1}]$
Rest	$1.21\cdot 10^{-6}$	$1.11\cdot 10^{-6}$
Exercise	$4.07\cdot 10^{-6}$	$3.72\cdot 10^{-6}$

## Data Availability

Data will be made available on reasonable request.
